# Central venous pressure and acute kidney injury in critically ill patients with multiple comorbidities: a large retrospective cohort study

**DOI:** 10.1186/s12882-022-02715-9

**Published:** 2022-02-28

**Authors:** Runlu Sun, Qi Guo, Junjie Wang, Yaoyao Zou, Zhiteng Chen, Jingfeng Wang, Yuling Zhang

**Affiliations:** 1 Department of Cardiology, Sun Yat-sen Memorial Hospital, Sun Yat-sen University, No. 107, the West Yanjiang Road, Yuexiu District, Guangzhou, 510120 China; 2 Guangdong Province Key Laboratory of Arrhythmia and Electrophysiology, Sun Yat-sen Memorial Hospital, Sun Yat-sen University, Guangzhou, China; 3 Department of Rheumatology, Sun Yat-Sen Memorial Hospital, Sun Yat-Sen University, Guangzhou, China

**Keywords:** Central venous pressure, Acute kidney injury, KDIGO stage, Critically ill patients

## Abstract

**Background:**

Given the traditional acceptance of higher central venous pressure (CVP) levels, clinicians ignore the incidence of acute kidney injury (AKI). The objective of this study was to assess whether elevated CVP is associated with increased AKI in critically ill patients with multiple comorbidities.

**Methods:**

This was a retrospective observational cohort study using data collected from the Medical Information Mart for Intensive Care (MIMIC)-III open-source clinical database (version 1.4). Critically ill adult patients with CVP and serum creatinine measurement records were included. Linear and multivariable logistic regression were performed to determine the association between elevated CVP and AKI.

**Results:**

A total of 11,135 patients were enrolled in our study. Critically ill patients in higher quartiles of mean CVP presented greater KDIGO AKI severity stages at 2 and 7 days. Linear regression showed that the CVP quartile was positively correlated with the incidence of AKI within 2 (*R*^*2*^ = 0.991, *P* = 0.004) and 7 days (*R*^*2*^ = 0.990, *P* = 0.005). Furthermore, patients in the highest quartile of mean CVP exhibited an increased risk of AKI at 7 days than those in the lowest quartile of mean CVP with an odds ratio of 2.80 (95% confidence interval: 2.32–3.37) after adjusting for demographics, treatments and comorbidities. The adjusted odds of AKI were 1.10 (95% confidence interval: 1.08–1.12) per 1 mmHg increase in mean CVP.

**Conclusions:**

Elevated CVP is associated with an increased risk of AKI in critically ill patients with multiple comorbidities. The optimal CVP should be personalized and maintained at a low level to avoid AKI in critical care settings.

## Background

Acute kidney injury (AKI) is a common complication in critically ill patients and has high morbidity and mortality [1]. Systemic and renal perfusion noticeably determines the development of AKI. However, optimal hemodynamic indicators of the risk of AKI have not been identified [2]. Although elevated fluid volume improves renal perfusion, aggressive fluid loading may lead to elevated central venous pressure (CVP). Given the traditional acceptance of higher CVP levels [3, 4], clinicians ignore elevated CVP, and the incidence of AKI is potentially interlaced.

CVP, a local hemodynamic parameter, reflects intravascular volume and is determined by the interaction between venous return and cardiac function [5]. Therefore, CVP is generally used for bedside assessment of volume status and responsiveness in critically ill patients [6]. Nonetheless, the validity of CVP in critical care settings has recently been challenged [7]. Based on the rationale provided by the Starling curves and Guyton theory on cardiac function [8], elevated CVP may increase venous pressure and decrease renal perfusion pressure, which further contributes to AKI. However, in critically ill patients with multiple comorbidities, including sepsis, heart failure, arrhythmias, hypertension, diabetes or others, the association between elevated CVP and AKI remains unclear.

Until recently, studies have shown inconsistent conclusions about the association of CVP and AKI in critically ill patients [9–11]. Herein, we sought to characterize the association of elevated CVP and AKI in critical care settings using the large, public, deidentified clinical database Medical Information Mart for Intensive Care (MIMIC)-III [12]. Specifically, we hypothesized that elevated CVP is associated with an increased incidence of AKI in critically ill patients with multiple comorbidities.

## Methods

### Data source

We conducted a large-scale, single-center, retrospective cohort study using data collected from the MIMIC-III open source clinical database (version 1.4), which was developed and maintained by the Massachusetts Institute of Technology, Philips Healthcare, and Beth Israel Deaconess Medical Center [12]. One author (Qi Guo) obtained access to the database and was responsible for data extraction (certification number: 25233333). Information derived from the 61,532 electronic medical records of critically ill patients admitted to intensive care units (ICUs) between 2001 and 2012 was included in this free, accessible database. The database was approved for research use by the Institutional Review Boards of the Massachusetts Institute of Technology and Beth Israel Deaconess Medical Center, and studies using the database were granted a waiver of informed consent.

### Study population

All patients in the database were screened according to the following inclusion criteria for this study: (1) adults (≥18 years of age at ICU admission); (2) ICU stay ≥1 day; and (3) for patients with multiple ICU stays, only the data for the first stay were considered. Patients with censored age, no CVP records, or no creatinine records were excluded.

### Variables

CVP, creatinine, and urine output records during the ICU stay were extracted. Other day 1 ICU measurement records were also extracted, including age, sex, weight, blood pressure and admission illness scores (the Simplified Acute Physiology Score (SAPS) [13] and the Sequential Organ Failure Assessment (SOFA) score) [14]. Moreover, data on the use of vasopressors, inotropes, sedatives, diuretics, invasive mechanical ventilation, and comorbidities, including sepsis, congestive heart failure (CHF), arrhythmias, hypertension, diabetes, chronic renal failure and cancer, were extracted from the database. In this study, vasopressors included norepinephrine, epinephrine, phenylephrine, vasopressin, and dopamine. Inotropes included dobutamine and milrinone. Chronic renal failure was defined as abnormalities of kidney structure or function (estimated glomerular filtration rate (eGFR) < 60 ml/min/1.73m^2^), present for more than 3 months, with implications for health [15]. Cardiac surgery recovery unit (CSRU) was used as variable to indicate the ICU type one patient stayed was CSRU. The comorbidities were determined from the International Classification of Disease, 9th Edition, Clinical Modification codes.

### Exposure

The primary exposure was the mean CVP during the first 7 days after ICU admission. We divided the mean CVP into four levels according to interquartile range as follows: quartile 1, CVP ≤ 8.29 mmHg; quartile 2, 8.29 < CVP ≤ 10.64 mmHg; quartile 3, 10.64 < CVP ≤ 13.20 mmHg; and quartile 4, CVP > 13.20 mmHg.

### Outcomes

The primary outcome was the odds of 2-day and 7-day AKI after ICU admission. We defined AKI by serum creatinine based on the KDIGO criteria [16]. AKI was categorized as Stage 1 if there was a 1.5–1.9 times serum creatinine increase from baseline, a 0.3 mg/dL serum creatinine increase or a urine output < 0.5 ml/kg/h for 6–12 h. Stage 2 was when there was a 2.0–2.9 times serum creatinine increase from baseline or a urine output < 0.5 ml/kg/h for ≥12 h, and Stage 3 was when there was a ≥ 3 times serum creatinine increase from baseline or a ≥ 4.0 mg/dL serum creatinine increase or urine output < 0.3 ml/kg/h for ≥24 h. The first serum creatinine record measured on ICU Day 1 was considered the “baseline”.

We calculated the CVP fluctuation within the first 2 days as follows: (mean CVP on the second day – mean CVP on the first day)/mean CVP on the first day. Patients were divided into 3 CVP trend groups: decreasing trend (fluctuation ≤ − 10%), increasing trend (fluctuation ≥10%), and stable trend (− 10% < fluctuation< 10%). Among the study population, 7397 patients suffered continuous CVP monitoring within the first 2 days. The association between this CVP trend group and AKI outcome was then evaluated for these patients.

Adjusted variables included age, male sex, weight, CSRU, ventilation use, vasopressor use, inotropes use, sedative use, diuretic use, SAPS score, SOFA score, sepsis, CHF, arrhythmias, hypertension, diabetes, chronic renal failure, cancer, systolic blood pressure (SBP), and diastolic blood pressure.

The association between CVP and AKI was further evaluated in patients using mechanical ventilation. To evaluate whether ventilation parameter influence our results, models were adjusted for age, male sex, weight, CSRU, ventilation use, vasopressor use, inotropes use, sedative use, diuretic use, SAPS score, SOFA score, sepsis, CHF, arrhythmias, hypertension, diabetes, chronic renal failure, cancer, SBP, diastolic blood pressure, and positive end-expiratory pressure.

### Statistical analysis

Normally distributed continuous variables are presented as the mean ± standard deviation, whereas nonnormally distributed data are presented as the median (interquartile range). Categorical variables are presented as numbers (percentages). Baseline characteristics were stratified by quartiles of mean CVP during the first 7 days after ICU admission. Baseline data were compared using the analysis of variance test or rank-sum test, as appropriate, for continuous variables, and the chi-square test was used for categorical variables. We performed linear and logistic regression to compute odds ratios (ORs) for the association of mean CVP with the odds of AKI.

All statistical analyses were performed using SPSS software (version 23.0, IBM, New York, USA) and R software (version 3.6.3, R Foundation for Statistical Computing, Vienna, Austria). A *P* value < 0.05 was considered statistically significant.

## Results

### Baseline characteristics

**Fig. 1:**
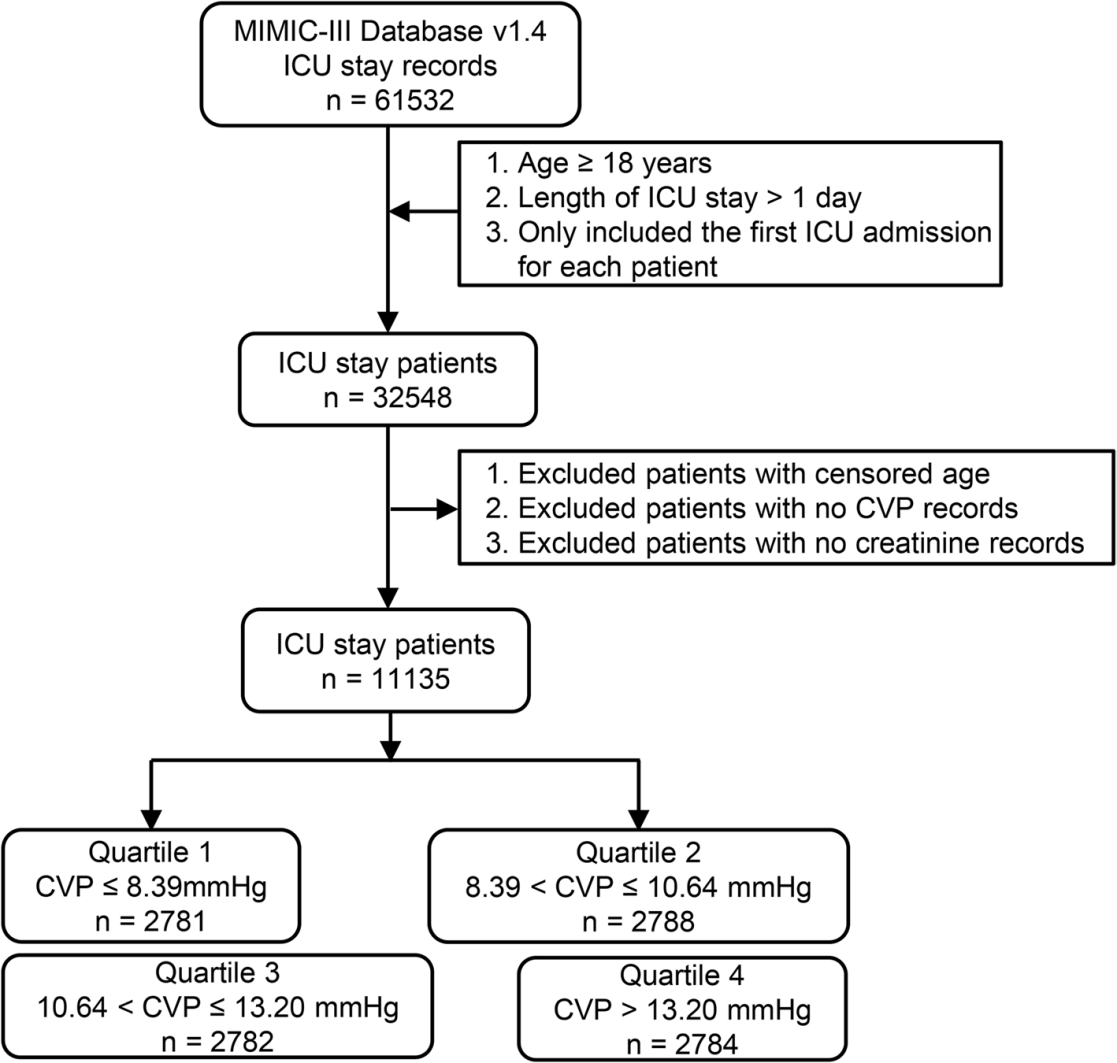
Flow chart of enrolled subjects. A total of 11,135 subjects were enrolled in our study. All enrolled subjects were divided into 4 groups based on CVP quartiles. ICU, intensive care unit; CVP, central venous pressure

Among the 61,532 ICU admissions in the MIMIC-III v1.4 database, 11,135 patients were enrolled in our study based on the inclusion and exclusion criteria in the Methods section (Fig. 1). The number of patients in each CVP quartile was approximately 2780. The mean CVP in each quartile was 6.7 ± 1.3 mmHg, 9.5 ± 0.6 mmHg, 11.9 ± 0.7 mmHg, and 16.0 ± 3.2 mmHg in the lowest to highest quartiles, respectively. Interestingly, patients in higher quartile of mean CVP had lower urine output, higher creatinine and lower eGFR. Also, patients in the highest quartile of mean CVP presented the greatest weight and highest SAPS and SOFA scores. Furthermore, patients with the highest quartile of mean CVP were likely to be treated with vasopressors and diuretics and more likely to have comorbidities of sepsis, CHF, arrhythmia, hypertension and chronic renal failure (Table 1).

**Table 1:** Characteristics of the enrolled subjects based on CVP quartiles

	CVP				
Variables	Quartile 1	Quartile 2	Quartile 3	Quartile 4	*P*
n	2781	2788	2782	2784	
CVP, mmHg	6.7 ± 1.3	9.5 ± 0.6	11.9 ± 0.7	16.0 ± 3.2	< 0.001^a,b,c,d,e,f^
Urine output, ml	2080.0(1340.0–2980.0)	1961.0(1320.0–2768.0)	1850.0(1195.8–2665.0)	1535.0 (870.0–2365.0)	< 0.001^a,b,c,d,e,f^
Creatinine, mg/dl	0.8 (0.6–1.1)	0.9 (0.7–1.2)	0.9 (0.7–1.3)	1.0 (0.8–1.6)	< 0.001^a,b,c,d,e,f^
eGFR, ml/min/1.73m^2^	87.7(63.1–101.2)	83.7(56.7–97.4)	80.8(52.1–96.6)	69.0 (39.1–91.7)	< 0.001^a,b,c,d,e,f^
Age, years	65.0 ± 15.1	65.8 ± 14.1	65.4 ± 14.1	64.1 ± 14.1	< 0.001^a,c,e,f^
Male	1729 (62.2)	1773 (63.6)	1691 (60.8)	1650 (59.3)	0.007^e^
Weight, kg	76.0 ± 21.6	81.4 ± 18.8	85.3 ± 21.1	90.9 ± 25.0	< 0.001^a,b,c,d,e,f^
SBP, mmHg	116.5 ± 14.2	113.9 ± 12.5	113.0 ± 12.6	110.3 ± 12.3	< 0.001^a,b,c,d,e,f^
DBP, mmHg	57.7 ± 8.6	57.5 ± 7.9	57.9 ± 8.1	57.9 ± 8.3	0.268
SAPS	20.0 (17.0–22.0)	20.0 (17.0–23.0)	20.0 (18.0–23.0)	21.0 (18.0–24.0)	< 0.001^a,b,c,e,f^
SOFA	4.0 (3.0–6.0)	5.0 (3.0–7.0)	5.0 (4.0–8.0)	7.0 (5.0–9.0)	< 0.001^a,b,c,d,e,f^
CSRU	1354 (48.7)	1563 (56.1)	1582 (56.9)	1374 (49.4)	< 0.001^a,b,e,f^
**Treatment**					
Vasopressor	1551 (55.8)	1872 (67.1)	1970 (70.8)	2054 (73.8)	< 0.001^a,b,c,d,e,f^
Inotropes	102 (3.7)	157 (5.6)	267 (9.6)	465 (16.7)	< 0.001^a,b,c,d,e,f^
Sedative	2003 (72.0)	2218 (79.6)	2291 (82.4)	2263 (81.3)	< 0.001^a,b,c,d^
Ventilation	2123 (76.3)	2336 (83.8)	2430 (87.3)	2406 (86.4)	< 0.001^a,b,c,d,e^
Diuretic	209 (7.5)	295 (10.6)	330 (11.9)	432 (15.5)	< 0.001^a,b,c,e,f^
**Comorbidities**					
Sepsis	714 (25.7)	805 (28.9)	843 (30.3)	1207 (43.4)	< 0.001^a,b,c,e,f^
CHF	258 (9.3)	266 (9.5)	304 (10.9)	394 (14.2)	< 0.001^c,e,f^
Arrhythmias	268 (9.6)	267 (9.6)	299 (10.7)	411 (14.8)	< 0.001^c,e,f^
Hypertension	163 (5.9)	200 (7.2)	215 (7.7)	284 (10.2)	< 0.001^b,c,e,f^
Diabetes	682 (24.5)	819 (29.4)	868 (31.2)	883 (31.7)	< 0.001^a,b,c^
Chronic renal failure	210 (7.6)	247 (8.9)	256 (9.2)	380 (13.6)	< 0.001^c,e,f^
Cancer	137 (4.9)	92 (3.3)	72 (2.6)	68 (2.4)	< 0.001^a,b,c^

Quartile 1, CVP ≤ 8.39 mmHg; Quartile 2, 8.39 < CVP ≤ 10.64 mmHg; Quartile 3, 10.64 < CVP ≤ 13.20 mmHg; Quartile 4, CVP > 13.20 mmHg. *CVP* central venous pressure; *eGFR* estimated glomerular filtration rate; *CSRU* cardiac surgery recovery unit; *SAPS* simplified acute physiology score; *SOFA* Sequential Organ Failure Assessment; *CHF* congestive heart failure; *SBP* systolic blood pressure; *DBP* diastolic blood pressure. Superscripts: a indicates a significant difference in the comparison of quartile 1 vs. quartile 2, b for quartile 1 vs. quartile 3, c for quartile 1 vs. quartile 4, d for quartile 2 vs. quartile 3, e for quartile 2 vs. quartile 4, and f for quartile 3 vs. quartile 4

### Elevated CVP and AKI outcome at 2 and 7 days

**Fig. 2:**
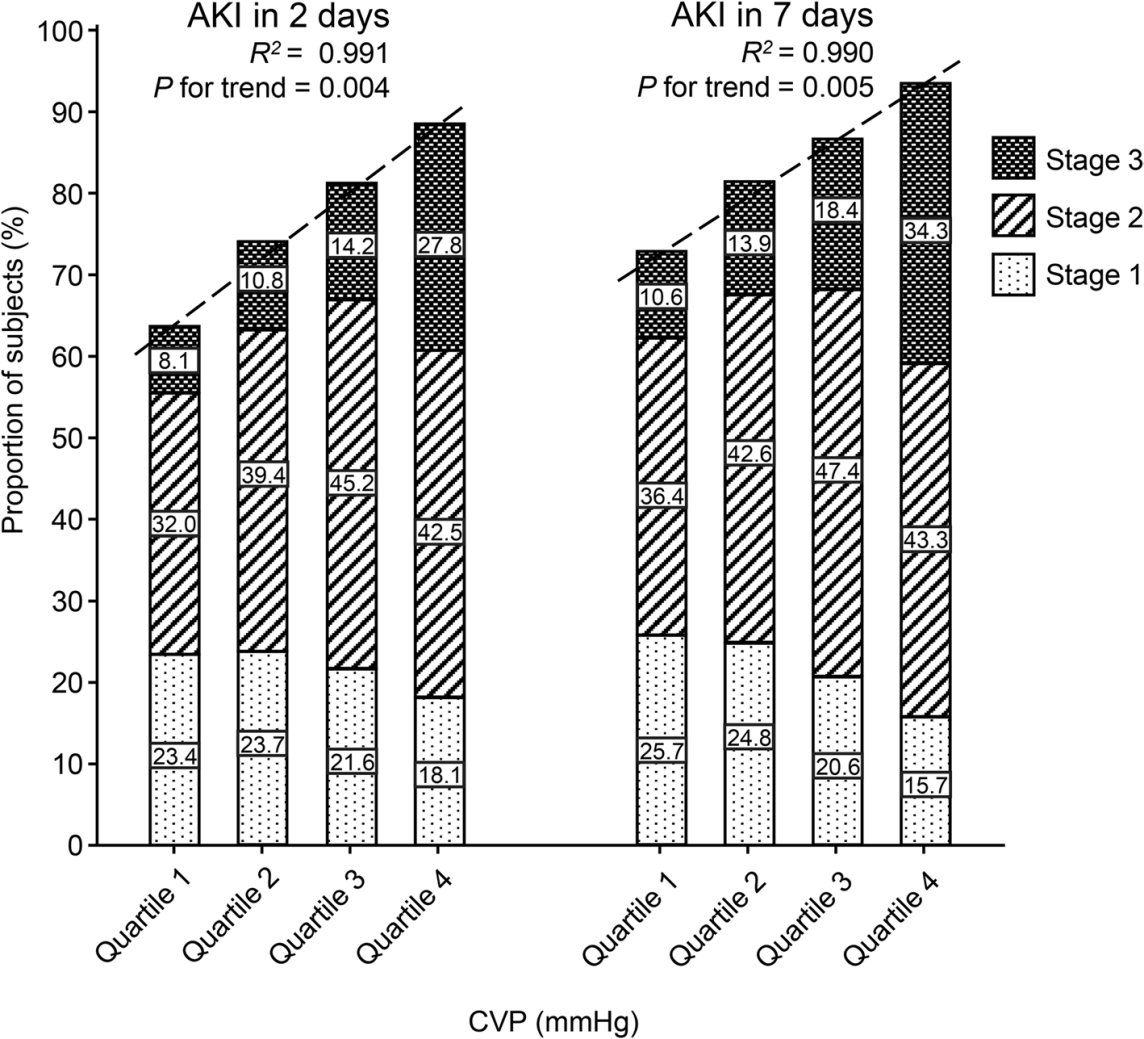
Proportion of AKI patients in different CVP quartiles and correlation between CVP quartile and AKI. The proportion of patients with different AKI severity stages is shown in each group with different CVP quartiles. AKI, acute kidney injury; CVP, central venous pressure

During the first 2 days and 7 days, 8544 and 9289 patients had AKI, respectively. The incidence of AKI at 2 days was greater among patients with higher CVP, ranging from 63.6% in patients with a mean CVP ≤ 8.39 mmHg (Quartile 1) to 88.4% in patients with a mean CVP > 13.20 mmHg (Quartile 4). A similar trend was detected between the incidence of AKI at 7 days and the mean CVP. Moreover, patients in the higher quartiles of mean CVP presented greater KDIGO AKI severity stages at 2 days and 7 days (Table 2). Additionally, linear regression showed that the CVP quartile was positively correlated with the incidence of AKI at 2 days (*R*^*2*^ = 0.991, *P* = 0.004) and 7 days (*R*^*2*^ = 0.990, *P* = 0.005) (Fig. 2).

**Table 2:** Association of CVP quartiles and AKI at 2 days or 7 days

	CVP				
	Quartile 1	Quartile 2	Quartile 3	Quartile 4	*P*
AKI in 2 days					
AKI	1768 (63.6)	2063 (74.0)	2251 (80.9)	2462 (88.4)	< 0.001^a,b,c,d,e,f^
Stage 1	652 (23.4)	662 (23.7)	600 (21.6)	504 (18.1)	< 0.001^c,e,f^
Stage 2	890 (32.0)	1099 (39.4)	1257 (45.2)	1184 (42.5)	< 0.001^a,b,c,d^
Stage 3	226 (8.1)	302 (10.8)	394 (14.2)	774 (27.8)	< 0.001^a,b,c,d,e,f^
AKI in 7 days					
AKI	2022 (72.7)	2267 (81.3)	2404 (86.4)	2596 (93.2)	< 0.001^a,b,c,d,e,f^
Stage 1	715 (25.7)	691 (24.8)	574 (20.6)	437 (15.7)	< 0.001^b,c,d,e,f^
Stage 2	1013 (36.4)	1188 (42.6)	1318 (47.4)	1205 (43.3)	< 0.001^a,b,c,d,e,f^
Stage 3	294 (10.6)	388 (13.9)	512 (18.4)	954 (34.3)	< 0.001^a,b,c,d,e,f^

KDIGO stage represents the severity of AKI according to the baseline measurement and the increase in serum creatinine or urine output. KDIGO Stage 1 was defined as a low severity of AKI, Stage 2 was defined as medium severity AKI, and Stage 3 was defined as high severity AKI. *CVP* central venous pressure; *AKI* acute kidney injury

**Fig. 3:**
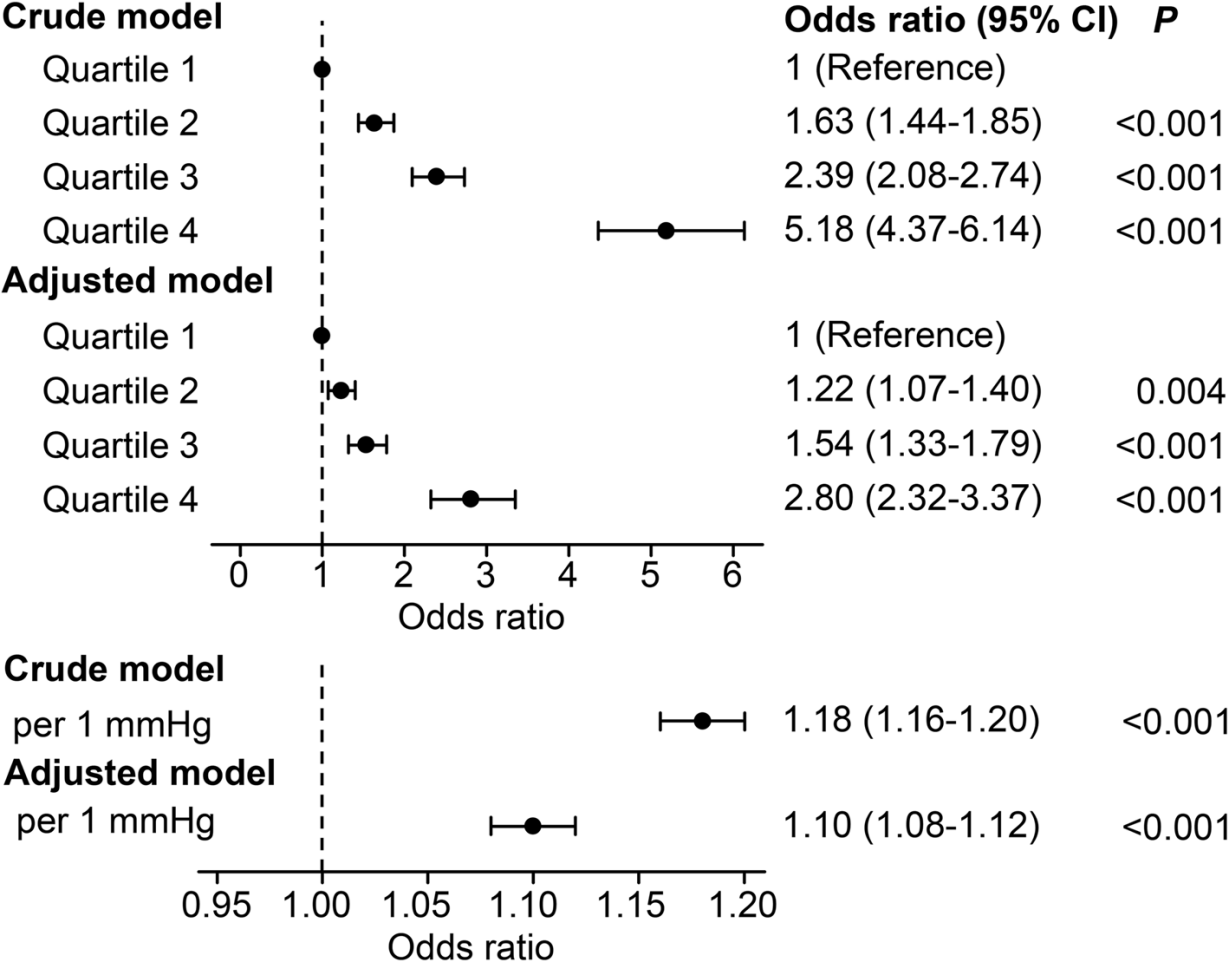
Odds ratios for AKI within 7 days associated with CVP in critically ill patients. For categorical variables, all odds ratios (95% CI) and *P* values were calculated compared with the lowest quartile. For continuous variables, odds ratios (95% CI) corresponded to a 1 mmHg increase in CVP. CVP, central venous pressure; AKI, acute kidney injury; CI, confidence interval

We further performed a logistic regression analysis to determine the association between quartiles of mean CVP and AKI outcomes in 7 days. For the crude model, patients in higher quartiles of mean CVP had a greater incidence of AKI at 7 days than those in the lowest quartile of mean CVP, ranging from OR = 1.63 (95% CI: 1.44–1.85) to OR = 5.18 (95% CI: 4.37–6.14). After adjustment, the mean CVP quartile remained a significant predictor of AKI at 7 days (Fig. 3). Furthermore, the odds of AKI were 1.18-fold (95% CI: 1.16–1.20) higher per 1 mmHg increase in mean CVP. After adjusting for demographics, treatments and comorbidities, the odds of AKI was 1.10 (95% CI: 1.08–1.12).

### Subgroup analysis by demographics, treatments and comorbidities

**Fig. 4:**
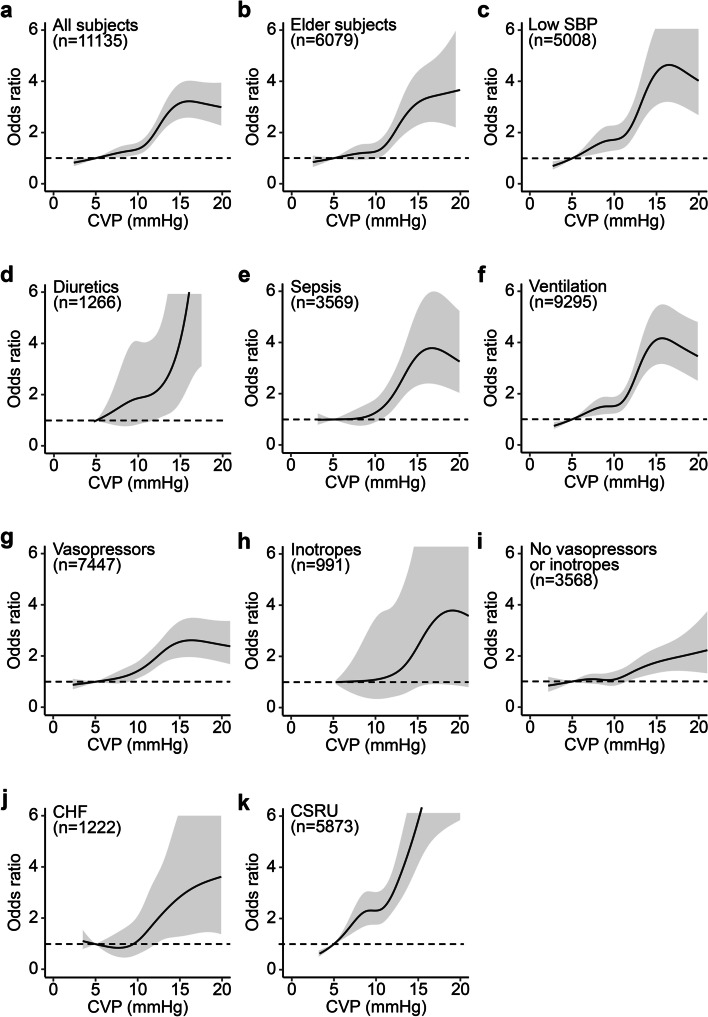
Odds ratios and 95% CI for AKI within 7 days associated with CVP in subgroups. Odds ratios (solid line) and 95% CI (gray area) for AKI associated with CVP in (**a**) all subjects, (**b**) subgroup with age > 65 years, (**c**) subgroup with SBP < 110 mmHg, (**d**) subgroup with use of diuretics, (**e**) subgroup with sepsis, (**f**) subgroup with use of ventilation, (**g**) subgroup with use of vasopressors, (**h**) subgroup with use of inotropes, (**i**) subgroup without use of any vasopressors or inotropes, (**j**) subgroup with CHF, and (**k**) subgroup in CSRU. The results were calculated using an adjusted restricted cubic spline model with a reference CVP of 5 mmHg (dotted line). CVP, central venous pressure; AKI, acute kidney injury; CSRU, cardiac surgery recovery unit; CHF, congestive heart failure; SBP, systolic blood pressure; CI, confidence interval

Next, we determined the association between mean CVP and AKI in subgroups of patients with an older age, low SBP and a history of cardiac surgery, patients treated with vasopressors, diuretics and ventilation, and patients with CHF and sepsis as comorbidities. Our data showed that CVP in all 11,135 subjects was positively correlated with AKI (Fig. 4a). Additionally, among 6079 elderly patients (age ≥ 65 years), patients with a mean CVP of greater than 11 mmHg had higher odds of AKI than those with a mean CVP of 5 mmHg (Fig. 4b). Moreover, 5008 subjects with SBP ≤ 110 mmHg and higher mean CVPs had higher odds of AKI (Fig. 4c). Additionally, a higher mean CVP (approximately CVP > 10 mmHg for diuretics and CVP > 8 mmHg for vasopressors) suggested an increased incidence of AKI in patients with use of diuretics, sepsis, use of ventilation, use of vasopressors (Fig. 4d & e & f & g). However, in subjects with use of inotropes, with no use of vasopressors or inotropes, with CHF, the mean CVP from 5 mmHg to 10 mmHg did not show significant correlation with AKI (Fig. 4h & i & j). Nevertheless, an elevated mean CVP was positively correlated with the incidence of AKI for patients in the CSRU (Fig. 4k).

**Fig. 5:**
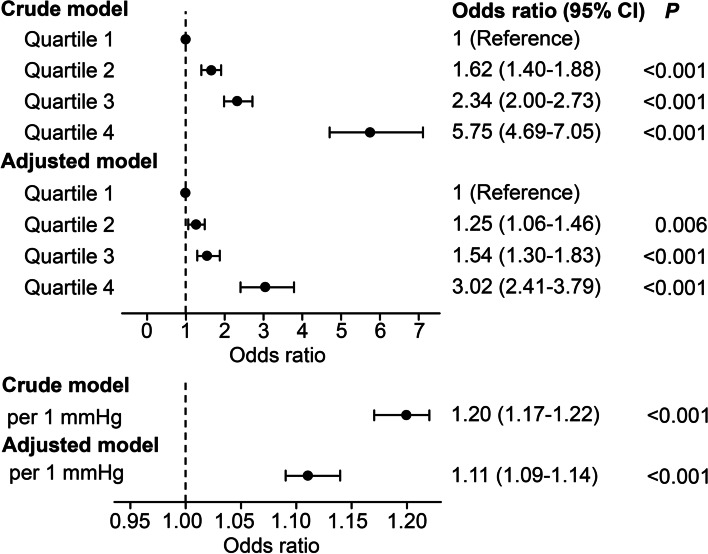
Association between CVP and AKI in subjects using ventilation. For categorical variables, all odds ratios (95% CI) and *P* values were calculated compared with the lowest quartile. For continuous variables, odds ratios (95% CI) corresponded to a 1 mmHg increase in CVP. CVP, central venous pressure; AKI, acute kidney injury; CI, confidence interval

To address the effect of the ventilation properties on the results, subgroup analyses in patients using invasive mechanical ventilation were performed. After adjustment for a series of variables, the CVP quartile 4 group showed a significantly higher risk of AKI than the CVP quartile 1 group (OR: 3.02, 95% CI: 2.41–3.79). The odds of AKI were 1.11 (95% CI: 1.09–1.14) times higher per 1 mmHg increase in mean CVP in this ventilation subgroup (Fig. 5).

### Association between CVP trend and AKI

Given that CVP was a dynamic parameter, we also investigated the association between CVP trend and AKI. In the crude model, both the decreasing trend group and increasing trend group showed significantly lower AKI risk than the stable trend group (*P* < 0.001). These associations were diminished after further adjustment (*P* > 0.05) (Table 3).

**Table 3:** Association between CVP trend and AKI at 2 days or 7 days

		Odds ratio	95% CI	*P*
Crude model	AKI in 2 days			
	Decreasing trend	0.865	0.752–0.996	0.043
	Stable trend	1 (Reference)		
	Increasing trend	0.933	0.810–1.076	0.342
	AKI in 7 days			
	Decreasing trend	0.808	0.682–0.958	0.014
	Stable trend	1 (Reference)		
	Increasing trend	0.782	0.660–0.927	0.005
Adjusted model	AKI in 2 days			
	Decreasing trend	0.909	0.780–1.060	0.224
	Stable trend	1 (Reference)		
	Increasing trend	1.083	0.928–1.265	0.312
	AKI in 7 days			
	Decreasing trend	0.842	0.701–1.012	0.067
	Stable trend	1 (Reference)		
	Increasing trend	0.887	0.739–1.066	0.202

Odds ratios (95% CI) were calculated compared with the stable trend group. *CVP* central venous pressure; *AKI* acute kidney injury; *CI* confidence interval

## Discussion

To our knowledge, this study is the first to evaluate the association between elevated CVP and AKI in critically ill patients with multiple comorbidities from a large-scale, public, deidentified clinical database (MIMIC-III). The three principal findings are summarized as follows: (1) Elevated mean CVP is associated with an increased risk of AKI in critically ill patients; (2) A 1 mmHg increase in CVP increases the odds of AKI in critically adult patients; (3) For critically ill patients with an older age, low SBP, a history of treatment with diuretics, vasopressors and ventilation, comorbidities of sepsis, or in the CSRU, the mean CVP level remained a significant predictor of AKI.

Clinicians use CVP as a measure of venous congestion in critically ill patients. Indeed, CVP has been censured as an unusable measurement of venous congestion due to other variables that can alter its value, including the relative height of the intravenous catheter to that of the barometer, artificial ventilation patterns, and changes in cardiac performance [17]. Despite the valid criticism, CVP is a potentially useful measure of venous congestion when we recognize its fluctuations due to the above variables [18].

The association between CVP and AKI has been determined previously [19], and a higher CVP is associated with poorer kidney function [9, 11, 20]. However, these findings were restricted to patients receiving diuretics, undergoing cardiac surgery and experiencing heart failure. Therefore, the association between elevated CVP and AKI remains unclear in critically ill patients overall after adjustment for demographics, treatments and comorbidities. Legrand et al. found a linear relationship between CVP and the incidence of AKI [21], and a meta-analysis demonstrated that a 1 mmHg increase in CVP increases the odds of AKI in critically adult patients [10], which is consistent with the findings of our study. In particular, in subgroups of patients with older age, low SBP and cardiac surgery, those undergoing treatment with vasopressors, diuretics and ventilation and those with sepsis as a comorbidity, we found that elevated CVP was still correlated with the odds of AKI. However, in subjects with CHF or with use of inotropes, a trend was not found possibly due to the limited sample size.

A more thorough understanding enables revaluation of the interaction between CVP and AKI. As an indicator of cardiac preload and renal afterload, CVP is determined by the interaction between cardiac function and venous return [22, 23]. Decreased renal function would lead to more liquid retention and further increase the CVP [17]. On the other hand, based on Guyton’s theory, cardiac output equals venous return, and venous reflux is dependent on the mean circulatory filling pressure (MCFP) and CVP gradient [8]. Specifically, extra fluid only increases CVP and tissue edema but does not significantly increase end-diastolic volume or stroke volume. When CVP was increased or MCFP was decreased, venous reflux was decreased; in contrast, venous reflux was increased when CVP was decreased or MCFP was increased [24, 25]. Therefore, lower CVP is necessary to ensure venous reflux and cardiac output when MCFP is in the flat part of the Starling curve. In fact, a healthy individual has a relatively low CVP [26]. According to this theory, it is hypothesized that a high CVP is transmitted backward, increasing renal venous pressure, reducing renal perfusion pressure and increasing renal venous congestion, further leading to AKI [19, 27].

In septic patients, CVP was reported to be associated with AKI risk even after adjustment for positive end-expiratory pressure [21]. Likewise, our study showed that the positive association between CVP and AKI risk persisted after adjustment for positive end-expiratory pressure in subgroup with use of ventilation. Both CVP and positive end-expiratory pressure were shown to be independently associated with worsening of renal function [28]. Meanwhile, several studies demonstrated that the increase of positive end-expiratory pressure could led an increase in CVP [29, 30]. Taken together, high positive end-expiratory pressure might involve with AKI, at least partly, by increasing CVP. Nevertheless, the mediating effect of CVP deserves further investigations.

Our study was based on data extracted from electronic medical records in MIMIC-III v1.4 [12], a large open clinical database, allowing precise research on the effects of an elevated CVP load. The use of database technologies and statistics played a critical role in achieving the meaningful conclusion of the present study. Additionally, this study has several limitations. First, this study is imperfect due to its retrospective nature and the source of the data used. Given the retrospective cohort study design, it is impossible to identify a causal link between AKI and CVP. Hence, no valid causal relationship can be established. Next, preadmission serum creatinine determinations were unavailable, and some patients may have already developed AKI on admission. Thus, the odds of AKI may have been underestimated during the ICU stay. Finally, although some predictors of disease severity were included in our study and adjusted analysis confirmed the association between elevated CVP and the incidence of AKI, the results may be affected by other confounding factors associated with AKI. Additional prospective studies should be conducted to evaluate these parameters and the potential effect of elevated CVP load.

## Conclusions

In conclusion, this study found that elevated mean CVP is associated with an increased risk of AKI in critically ill patients with multiple comorbidities. Individualizing CVP measurements and maintaining a low CVP should be encouraged to avoid unnecessary renal damage.

## Data Availability

The data that support the findings of this study are available from the Institutional Review Boards of the Massachusetts Institute of Technology and Beth Israel Deaconess Medical Center, but restrictions apply to the availability of these data, which were used under license for the current study and are therefore not publicly available. Data are however available from the authors (Prof. Yuling Zhang, E-mail: zhyul@mail.sysu.edu.cn or Prof. Jingfeng Wang, E-mail: wjingf@mail.sysu.edu.cn, Dr. Runlu Sun, E-mail: sunrlu@mail.sysu.edu.cn or Dr. Qi Guo, E-mail: guoq69@mail.sysu.edu.cn) upon reasonable request and with permission of the Institutional Review Boards of the Massachusetts Institute of Technology and Beth Israel Deaconess Medical Center.

## Abbreviations

AKI: Acute kidney injury
CHF: Congestive heart failure
CSRU: Cardiac surgery recovery unit
CVP: Central venous pressure
ICU: Intensive care unit
MIMIC: Medical Information Mart for Intensive Care
OR: Odds ratio
SAPS: Simplified Acute Physiology Score
SBP: Systolic blood pressure
SOFA: Sequential Organ Failure Assessment
